# Editorial Note on the 10-Year Anniversary of the International Journal of Disaster Risk Science: A Thank You Letter

**DOI:** 10.1007/s13753-020-00297-4

**Published:** 2020-08-28

**Authors:** Peijun Shi, Carlo Jaeger

**Affiliations:** 1 Academy of Disaster Reduction and Emergency Management, Ministry of Emergency Management and Ministry of Education, Faculty of Geographical Science, Beijing Normal University, Beijing, 100875 China; 2 Global Climate Forum, 10178 Berlin, Germany

This year marks the 10-year anniversary of the *International Journal of Disaster Risk Science* (*IJDRS*). On this occasion, as Editors-in-Chief, we are deeply grateful for the support and contribution of many who have enabled the growth of the journal over the past decade—contributing authors from many disciplines around the world, our reviewers who have volunteered their valuable time and expertise, dedicated editorial board members and our tireless and meticulous editorial team, and after all, you, millions of global readers who share the mission of making our world safer. Thank you all!

## How did It Start?

The impetus for the initiation of the *IJDRS* had to do with two major disaster events and two major scientific endeavors. The former symbolized the context and call for integrated disaster risk studies responsive to rapidly changing Chinese and global situations, whereas the later provided enabling conditions.

The two disaster events are the Wenchuan Earthquake and the global finical crisis. Occurring on 12 May 2008, the Wenchuan Earthquake was the most severe earthquake disaster in China since the Tangshan Earthquake of 1976, which took place right before the beginning of the opening up and reform period in China in the late 1970s. Thus, the Wenchuan Earthquake brought about broad reflections on the rapidly changing vulnerability and exposure to disaster risks over about 30 years of transformative socioeconomic changes in China. The disaster highlighted the critical importance of understanding the dynamic nature of vulnerability as well as the need for evolving disaster risk management capacities in close relation with the choices and pathways of development. In addition, the Wenchuan Earthquake also became the first major disaster event in China that had drawn worldwide attention and examination, and highlighted the need to bridge exchange and communication between disaster risk reduction communities in China and internationally.

Also in 2008, the spread of the global financial crisis shocked the world. Among its many facets, the global financial crisis exemplified the changing global risk landscape in the age of globalization and an increasingly interconnected world, and highlighted the systematic nature of disaster risk impacts, which has been revealed again with today’s ongoing Covid-19 pandemic.

Two major scientific endeavors launched at that time provided the enabling conditions for the creation of the journal. One was the establishment of the Hazard and Risk Science Base (HRSB, 2008–) at the Academy of Disaster Reduction and Emergency Management (ADREM), Beijing Normal University (BNU); the other was the launch of the then International Human Dimensions Programme on Global Environmental Change (IHDP) core science project—Integrated Risk Governance Project (IRG) (2008–2015; and Future Earth, 2015–2025)—the first of such that was initiated and led by China.

Funded by the Ministry of Education and the State Administration of Foreign Experts Affairs of China, the HRSB at BNU enabled construction of a platform that strengthened international collaboration and promoted the involvement of leading disaster risk experts around the world. To guide both educational disciplinary development as well as integrated disaster risk research at the HRSB, a Scientific Steering Committee that consisted of both international and Chinese experts was established. One of the earlier core recommendations from the Committee was to develop a scientific journal that would: (1) promote the integrated disaster risk studies that underpin and support integrated risk management and governance; (2) follow closely the development of the international disaster risk reduction agenda, and reflect and synthesize timely learning from major disaster risk events around the world; and (3) bridge and facilitate exchange and communication between disaster risk reduction (DRR) communities in China and globally.

The new journal recommendation also fitted well with the Science Plan of the IRG, since its implementation required an outlet for dissemination. With further strong endorsement from the Ministry of Civil Affairs—the ministry mainly responsible for managing natural hazard related disasters in China then^1^—and financial support from Beijing Normal University, the *International Journal of Disaster Risk Science* was born in 2010. The inauguration volume contained two issues.^2^

## The Ten Years of Growth

A decade later, we have witnessed many changes in the field. At the international and policy level, we saw the completion of the *Hyogo Framework for Action* as the global blueprint for disaster risk reduction efforts between 2005 and 2015, followed by the launch of the *Sendai Framework for Disaster Risk Reduction 2015*–*2030*. Numerous regional, national, and local initiatives on disaster risk reduction, innovative scientific research, development and broader application of new technologies, and growing stakeholder participation have come into being. The *IJDRS* has reflected the changes and the dynamics of the field by promoting and focusing on integrated and transdisciplinary disaster risk studies through theoretical, methodological, and case studies. We followed the Sendai Framework as it was launched, marked by the publication of the special issue on Analyzing the Sendai Framework for Disaster Risk Reduction in June 2015. We reported the Science and Technology Roadmap to Support the Implementation of the Sendai Framework for Disaster Risk Reduction 2015–2030. This year we also gathered views on the first five years of the Sendai Framework implementation in a special issue on Five Years of the Sendai Framework for Disaster Risk Reduction. We published a number of special issues and special sections, for example on: Disaster and Urbanism; The Usefulness of Resilience and Vulnerability for Disaster Risk Management; Health and Risks; Risk Governance, Assessment, and Economic Impacts; and Participatory Risk Governance, which all reflect the journal’s thematic focus. Highlighting the linkages and integration between development and DRR, we also published articles as the output of the series of Shenzhen International Symposium on Green Development and Integrated Risk Governance, just to name one example.

The journal has been indexed by the relevant main abstracting and indexing databases including Science Citation Index Expanded (SCIE), Scopus, GeoRef, Geobase, Chinese Science Citation Database (CSCD), among around 35 others. We saw a steady growth of annual submissions to the journal as well as over a quarter million downloads of the online articles in 2019. This year, the publication of the *IJDRS* has been expanded from quarterly to bimonthly to accommodate the growing number of contributions. All articles published are made freely and permanently accessible online immediately upon publication, without subscription charges or registration barriers, and also are free of article processing charges to authors owing to the support of BNU and the China Association for Science and Technology. Today, we are proud to see that the *IJDRS* is among the key scientific journals internationally in the field of disaster risk studies.

## Moving Forward

Looking back to the three objectives and expectations originally set forth by the HRSB Scientific Steering Committee, we see they have been instrumental to journal identity and remain the main core niches of the *IJDRS*. As the Hazard and Risk Science Base at BNU continues and *IJDRS* grows into its teen years, we see those needs that had stimulated and motivated the creation of the journal remain, if anything, even stronger—the need for integrated, inter- and transdisciplinary disaster risk studies, the need for translating science to policy and practice, the need to explore and enhance governance capacities for disaster risk reduction that are increasingly systemic in nature.

The coming decade is critical for the implementation of major global agreements of the Sendai Framework for Disaster Risk Reduction 2015–2030, the Paris Agreement on climate change, and the 2030 Agenda for Sustainable Development and sustainable development goals. Disaster risk studies must address the complex interactions between disaster risks and development in the context of a changing climate in which all dimensions of disaster risk—hazard, exposure, vulnerability, and the contextual environment—need to be examined within a comprehensive and systemic perspective.

The scope and focus of the *IJDRS* aligns well with the four priority areas identified by the Sendai Framework, from understanding the changing nature and dynamics of risks, to strengthening integrated risk governance capacity and investment, best practices and lessons learned for recovery and redevelopment, and build back better. In the coming decade, the journal will follow closely and contribute to the implementation of the Sendai Framework.

We will encourage the exploration of systemic and cascading risks. Nothing has illustrated more strongly the need for and urgency of understanding the systemic nature of global risk and of strengthening the collaborative risk governance capacity in a truly interconnected world than the current ongoing Covid-19 pandemic. We anticipate that the *IJDRS* will follow closely the development and learning derived from this very real global human tragedy.

We will continuously facilitate the shift of paradigms from managing disasters to managing risks as called for by the Sendai Framework, which requires fundamentally transforming the relationship between disaster risk and development, and facilitating development as a means to reduce risks rather than creating new sources of vulnerability and disaster risk. Preventing the creation of risk requires understanding the complex interactions between socioeconomic processes and the natural environment, thus the relationship between development and disaster risk, risk transition, as well as integrated DRR and climate action measures will remain among key focus areas for *IJDRS*.

Moving forward, we are determined to make the *IJDRS* a truly open platform for researchers, practitioners, and policymakers to share knowledge and learning, exchange perspectives, engage in debate so to improve international, regional, national, and local capacities for disaster risk analysis, management, and governance in seeking integral solutions for sustainable and equitable economic, social, and environmental development around the world.

**Peijun Shi and Carlo Jaeger**

**Editors-in-Chief, International Journal of Disaster Risk Science**

**15 August 2020, Beijing, China and Potsdam, Germany**

## Footnotes

1 The responsibility was shifted to the newly created Ministry of Emergency Management in 2018.

2 The inauguration volume is available at https://link.springer.com/journal/13753/1/1 and https://link.springer.com/journal/13753/1/2.

